# Insect Gut Bacteria Promoting the Growth of Tomato Plants (*Solanum lycopersicum* L.)

**DOI:** 10.3390/ijms232113548

**Published:** 2022-11-04

**Authors:** Krzysztof Krawczyk, Alicja Szabelska-Beręsewicz, Sebastian Wojciech Przemieniecki, Mateusz Szymańczyk, Aleksandra Obrępalska-Stęplowska

**Affiliations:** 1 Department of Virusology and Bacteriology, Institute of Plant Protection-National Research Institute, Władysława Węgorka 20, 60-318 Poznan, Poland; 2 Department of Mathematical and Statistical Methods, Poznań University of Life Sciences, 28 Wojska Polskiego St, 60-624 Poznan, Poland; 3 Department of Entomology, Phytopathology and Molecular Diagnostics, University of Warmia and Mazury in Olsztyn, Prawocheńskiego 17, 10-720 Olsztyn, Poland; 4 Department of Breeding and Agriculture Technology for Fibrous and Energy Plants, Wojska Polskiego 70B, 60-630 Poznan, Poland; 5 Department of Molecular Biology and Biotechnology, Institute of Plant Protection—National Research Institute, 20 Węgorka St, 60-318 Poznan, Poland

**Keywords:** plant growth promoting bacteria, bacterial consortium, *Diabrotica virgifera*, microbiome, gut bacteria, insects’ symbionts

## Abstract

**Simple Summary:**

A new sources of plant growth-promoting (PGP) bacteria are needed to increase the agricultural crops without increasing the usage of chemicals. The aim of this study was to test the hypothesis that insect-gut bacteria promote tomato plant growth. The insect gut bacteria were screened for the presence of PGP traits. The plants treated with bacterial consortium showed a significant increase in fruit yield, in both number of fruits (+41%) and weight of fruits (+44%). Our results showed that: (i) *D. virgifera* gut’s bacteria significantly promote the growth of tomato plants, and (ii) bacteria other than plant-related can be considered as PGP. It must be underlined that even though the insect gut bacteria were proven to stimulate the plant’s growth, their practical usage must be preceded by an examination of their influence on ecological and biological safety.

**Abstract:**

We investigated gut bacteria from three insect species for the presence of plant growth properties (PGP). Out of 146 bacterial strains obtained from 20 adult specimens of *Scolytidae* sp., 50 specimens of *Oulema melanopus*, and 150 specimens of *Diabrotica virgifera*, we selected 11 strains displaying the following: PGP, phosphate solubility, production of cellulase, siderophore, lipase, protease, and hydrogen cyanide. The strains were tested for growth promotion ability on tomato (*Lycopersicon esculentum*) plants. Each strain was tested individually, and all strains were tested together as a bacterial consortium. Tomato fruit yield was compared with the negative control. The plants treated with bacterial consortium showed a significant increase in fruit yield, in both number of fruits (+41%) and weight of fruits (+44%). The second highest yield was obtained for treatment with *Serratia liquefaciens* Dv032 strain, where the number and weight of yielded fruits increased by 35% and 30%, respectively. All selected 11 strains were obtained from Western Corn Rootworm (WCR), *Diabrotica virgifera*. The consortium comprised: *Ewingella americana, Lactococcus garvieae, L. lactis*, *Pseudomonas putida*, *Serratia liquefaciens*, and *S. plymuthica*. To our knowledge, this is the first successful application of *D. virgifera* gut bacteria for tomato plant growth stimulation that has been described.

## 1. Introduction

Tomato (*Solanum lycopersicum* L.) is the world’s second most important vegetable crop next to potato, and one of the most important crops in Poland. The total European Union tomato production is estimated at 180.8 million tons per year (Faostat database 2019: http://www.fao.org/faostat/en/#data/QC, accessed on 22 January 2020), while Poland’s production is at 917.8 thousand tons per year in 2019 (Faostat database 2019: http://www.fao.org/faostat/en/#data/QC, accessed on 22 January 2020). Continuously increasing human population and rising consumer awareness put great pressure on farmers to constantly increase cropping efficiency and quality. To meet this goal many chemical substances like mineral fertilizers and pesticides are commonly used in agriculture. However, their performance seems to have reached its limits of efficacy and can be destructive for both crops and soil ecosystems. A solution to this problem might be biopreparates, a specially prepared formula containing live or hibernated microorganisms or products of the microbial metabolism, promoting the plant’s growth, and allowing the number of chemicals used in crop protection to be reduced. Bacteria, as a group of microorganisms, are one of the main groups of interest in the production of biopreparates for plant growth-promotion.

Bacteria are part of the plant’s microbiome and form a complex network of relationships that affect nearly every aspect of plant growth. The use of bacteria to improve tomato production has a well-documented history. Endophytic and rhizosphere bacteria were used to increase tomato yield in the greenhouse, e.g., *Bacillus* sp. [1], *Serratia marcescens* [2], *Achromobacter piechaudii* [3], *Azospirillum brasilense*, *Gluconacetobacter diazotrophicus*, *Herbaspirillum seropedicae*, and *Burkholderia ambifaria* [4].

To be able to promote plant growth, bacteria need to effectively colonize all plant-related ecological niches, such as rhizosphere, epiderm, and internal plant tissues, to interact with the plant host. To do this, they need to exhibit biochemical and physiological features, such as motility, attachment, plant-polymer degradation, and evasion of plant defenses [5]. Motility and polysaccharide production were proven crucial in the colonization of plant rhizosphere by endophytic bacteria *Alcaligenes faecalis* and *Azospirillum brasilense* [6]. Additionally, dissolving inorganic phosphates, and the production of protease, lipase and chitinase enzymes facilitate rhizosphere colonization [7]. The production of indole acetic acid (IAA), as was shown for *Pseudomonas putida* applied to healthy mature tomato plants in hydroponic culture [8]. Among other biochemical traits that were reported to promote plant’s growth are siderophore production [9], including pyoverdine production [10], phosphate dissolving [11], organic acids, and 1-aminocyclopropane-1-carboxylic acid (ACC) deaminase production [12]. 

The improvement in tomato fruit yield was also achieved by using the environmental bacteria as a biological control agent. The root colonizing strain of *Pseudomonas fluorescens* and *P. chlororaphis* suppresses the *Fusarium oxysporum* infection through the production of the antibiotic phenazine-1-carboxamide [13]. The plant growth-promoting bacteria (PGPB) can be also used as a biological control against plant-pathogenic bacteria, suppressing the *R. solanacearum*-caused disease development [14].

The plant growth-promoting rhizobacteria (PGPR) are isolated mainly from the rhizosphere [15]. That is why, in general, as PGPB, we consider only the bacteria directly connected with plants, e.g., endophytic or rhizospheric. However, plant growth-promoting bacteria are present in niches other than those directly connected with plants like the rhizosphere or plant surface [16]. In fact, in nature, there is a whole continuum of bacteria that interact with plants, e.g., through the plant-insect relationship. All bacteria grown under the plant’s cuticle can be exudate on the plant surface as effusion or oozing. These bacteria can be transferred from plant to plant by insects, both mechanically, on their mouthparts during feeding, and in the gut of sap-drinking insects that are able to puncture the cuticle. Insect feeding is a significant factor in bacteria transfer between plants. The relationships between bacteria and insects have a very long history. Bacteria inhabit insects’ guts, thus affecting their fitness. The insect’s gut provides good conditions for bacterial conjugation and plasmid transfer, which makes it a perfect place for gene transfer, as was shown through genome analysis [17]. Bacteria-insect relationships are complex and among positive interactions involve both commensalism and mutualism [18]. The internal diversity of the insect microbiome is related to a variety of gut structures and compartments, various pH levels, redox potential, digestive enzymes [19,20], and insect food [21]. The studies show that there is a relationship between gut structure and variation of the microbiome. Insects with a simple digestive tract have less differentiated microbiota, whereas a more complex digestive tract usually means more diversified microbiota [20]. Most insects are connected with bacteria creating symbiotic relationships with their host or being able to influence various biological functions of their host [22]. Insects, sterilized from bacteria, are not able to develop and multiply in a normal way, while symbiotic bacteria are not able to survive without their host [23]. Bacteria also influence the defense mechanisms against pathogens and parasites, and understanding the relationship of insect-symbiont-pathogen may lead to less extensive use of insecticides [24].

Studies on animal genomes have shown that animals do not have a complete metabolic system, and therefore, thanks to the community of gut microbes, they can extract the maximum amount of nutrients from their food [25]. Intestinal bacteria can thrive in a hostile environment, for example, and withstand extreme pH. Their reproductive process is faster in the gut than in vitro [26]. Detailed studies of gut bacteria were carried out on termites, beetles [27], and flies [28], while the most well-known group is Lepidoptera insects, which are phytophages and can digest cellulose [29]. The insect’s intestinal microbiome influences the effectiveness of plant defense mechanisms [30], and it can have a synergistic effect (increasing the defense effectiveness) or antagonistic (reducing the effectiveness) [31]. Studies have shown that different types of insect gut bacteria are closely related to the rhizosphere, phyllosphere, and soil bacteria [32]. The population of microbes in the insect’s intestine may be more abundant and more diverse than in the phyllosphere [33]. Many insect species are believed to obtain their microbiota from the environment [17,34]. Insect intestines may be a potential niche for the isolation of PGP bacteria, which may increase plant growth and mineral absorption. An example is the isolation of bacteria from the intestines of *Plutella xylostella* larvae. Three strains of bacteria were isolated: *Acinetobacter* sp., *Pseudomonas* sp., and *Serratia* sp. It was found that they all responded to phosphorus and zinc depletion. These bacteria influence various solubilization reactions, such as chelation or conversion of insoluble to soluble phosphorus, which can affect the growth of soybean in phosphorus-poor soil [35]. In a study by [32], eight bacteria isolated from the intestines of redfish (*Plutella xylostella*) larvae showed PGPB features. The isolates were able to fix nitrogen and produce salicylic and indole-3-acetic acids (IAA).

As shown above, the connections between insect and plant microbiota have started to be uncovered. Interactions between plant growth-promoting rhizobacteria, foliar-feeding insects and higher trophic levels including the roles of these bacteria in altering plant chemical defenses against foliar insects, changing plant-associated microbial community, and shaping plant-insect natural enemy multi-trophic interactions have been described [36]. The role of rhizobacteria in the control of pest insects in agriculture was also characterized [37].

In light of the research cited above, we decided to test the hypothesis that insect-gut bacteria promote tomato plant growth. One of the important prerequisites was the fact that we were able to isolate the PGP bacteria *Lactococcus lactis* and *Pseudomonas* sp. from the gut of *Oulema melanopus*, *Diabrotica virgifera*, and *Scolytidae* sp. This work aims to screen the insect gut bacteria for the presence of PGP traits described above, and to develop and test both the bacterial consortium and its strains, against the PGP effect on tomato plants, which would be assessed in comparison with the control group.

## 2. Results

### 2.1. Characteristics of Bacterial Strains

In general, 146 bacterial strains obtained from 20 adult specimens of *Scolytidae* sp., 50 specimens of *Oulema* sp. and 150 specimens of *Diabrotica virgifera* were screened for PGP properties. Of this group, 11 strains were selected for further research (Table 1). None of the 11 selected strains showed antagonistic properties against each other. All 11 strains endure glycerol preservation at −20 °C, and showed simultaneously a minimum 3 of PGP properties, assessed in vitro. The biochemical properties of the 11 isolates are presented in Table 1. All selected 11 isolates were obtained from the gut of *D. virgifera*.

The consortium comprised 11 selected strains belonging to six bacterial species: *Ewingella americana* (one strain), *Lactococcus garvieae* (one strain) *L. lactis* (one strain), *Pseudomonas* sp. (one strain), *Pseudomonas putida* (one strain), *Serratia plymuthica* (one strain), *S. liquefaciens* (four strains), and *Serratia* sp. (one strain), (Table 1). After bioreactor’s incubation three out of six species were recovered: *S. liquefaciens* (43% ~ 2.4 × 10^10^ cfu/mL), *S. plymuthica* (42% ~ 2.37 × 10^10^ cfu/mL) and *P. putida* (15% ~ 8.4 × 10^9^ cfu/mL). This result was expected because seven out of eleven strains of the consortium were *Serratia* sp. In addition, after the experiment, the soil was investigated and the very same three species: *S. liquefaciens* (4 × 10^8^ cfu/mL), *S. plymuthica* (3.1 × 10^9^ cfu/mL) and *P. putida* (2.6 × 10^9^ cfu/mL) were recovered.

### 2.2. The Greenhouse Test

**Figure 1:**
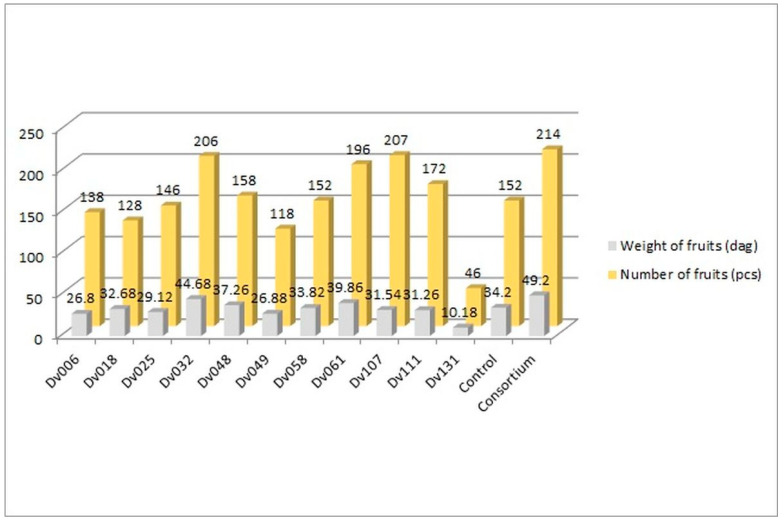
Comparison of the amount and weight of tomatoes from all 13 treatments.

**Figure 2:**
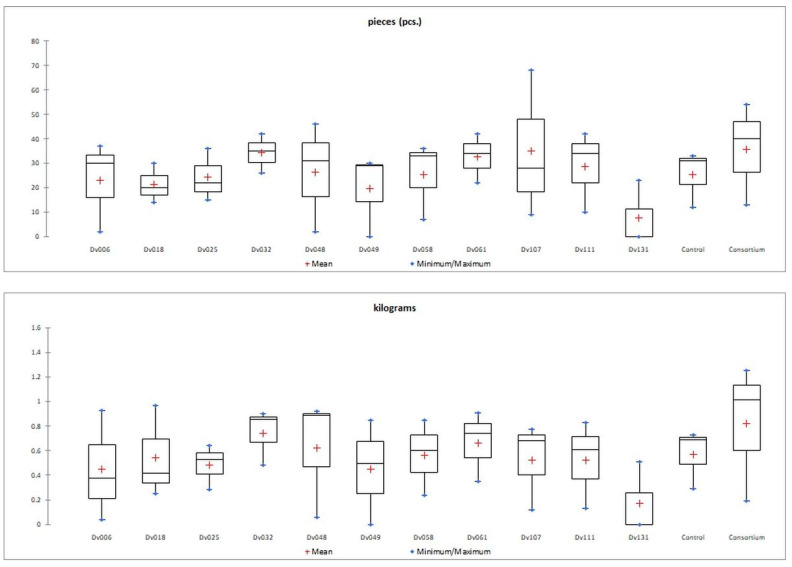
Graphical presentation (box-plot) of the distribution of a statistical feature of quantity (pieces) and mass (kilograms) of tomato fruit yield in each treatment.

**Figure 3:**
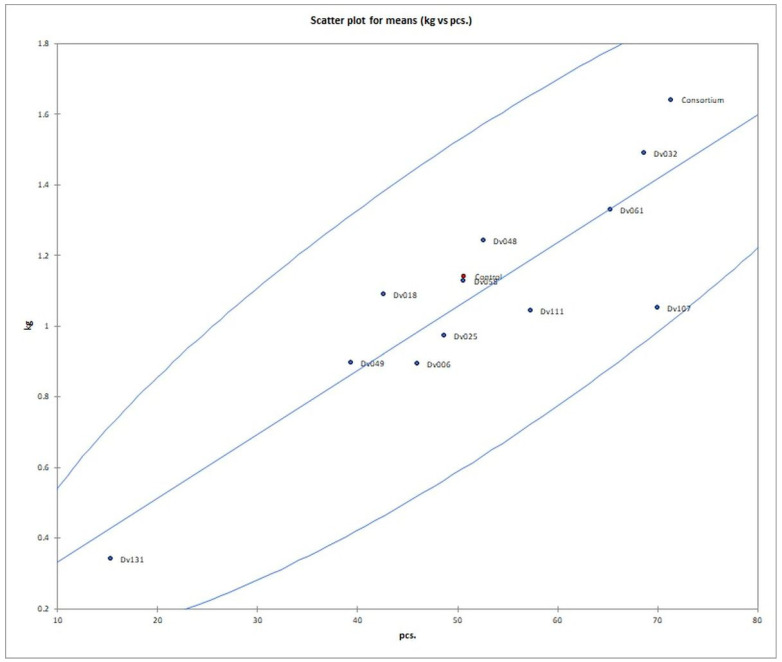
Scatter plot of number and mass yield of tomato fruits in each treatment. Red dot (Control) means negative control.

The greenhouse experiment was performed to assess the influence of bacteria on tomato yield. In total, 13 treatments were tested, including negative control, consortium, and each of the 11 strains comprising the consortium (Figure 1, Figure 2 and Figure 3). The plants in all treatments were grown in the soil until the fruits appeared. The crops were harvested continuously, counted, and weighed until no new fruits were available. The results showed an increase in both the number and weight of fruits, in comparison to the control group (Figure 1).

The number of fruits after consortium treatment was 41% (*p* = 0.004947) higher in comparison to the control group (Control), and the total mass of yielded fruits increased by 44% (*p* = 0.005165). The second highest yield was obtained for treatment with strain Dv032, where the number and mass of yielded fruits increased by 35% (*p* = 0.003308) and 30% (*p* = 0.004175), respectively, in comparison to the control group (Control) (Figure 1). The significant increase in tomato yield for both the number and the mass of fruit of bacteria-treated tomato plants is visualized by the bar graph (Figure 1). Results of statistical analysis of the quantity and mass of the yield of tomato fruits are presented using a box-plot (Figure 2) and scatter plot (Figure 3). As an addition, the phenotype of tomato plants was presented in photographs, in the Supplementary Materials (Figure S1).

### 2.3. Tomato Plants’ Growth Indexes

**Figure 4:**
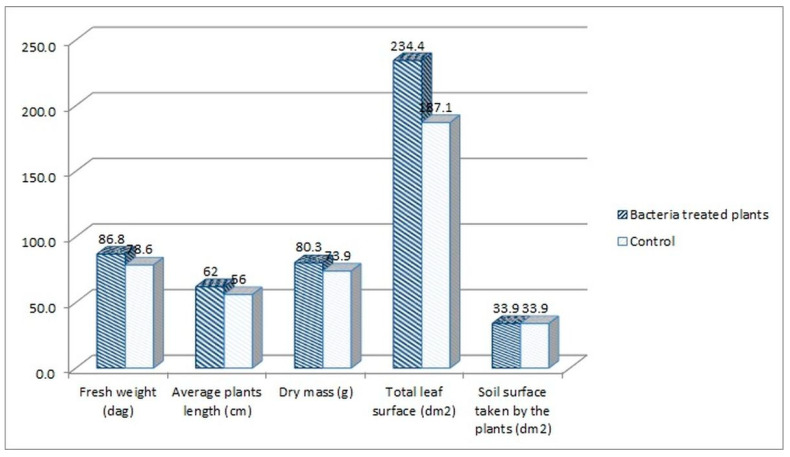
The parameters measured for the consortium-treated tomato plants in comparison to the control group, tomato plants treated only with water.

The following plant growth indexes: leaf area index (LAI), net assimilation rate (NAR) and crop growth ratio (CGR), were assessed for the tomato plants treated with the consortium bacteria, as the best performing group in the experiment. The indexes were calculated based on the measurement of plants’ fresh weight, average length, dry mass, and leaf surface. When comparing the consortium-treated tomato plants with the water-treated control group, higher parameters were noted for the consortium-treated plants. The fresh weight increased by 10.47%, the average length by 10.71%, the dry mass by 8.66%, and the leaf surface by 25.28% in comparison to the control (Figure 4).

**Figure 5:**
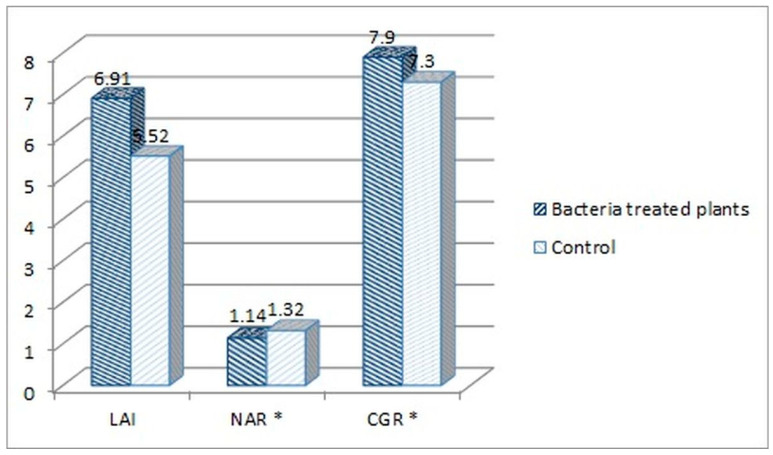
Comparison of the plant growth indexes of the consortium-treated tomato plants and water-treated control group. Calculated indexes: leaf area index (LAI), netto assimilation rate (NAR), and crop growth ratio (CGR) (* The NAR and CGR original values were multiplied both by 100, for better visualization on the chart).

Based on those results (Figure 4), the LAI, NAR and CGR indexes were calculated for both consortium-treated and water-treated control plants (Figure 5).

LAI index mathematically describes the foliage density by a relation of the total leaf surface of the plant to the soil surface on which the plant grew. The LAI index was higher for the consortium-treated tomato plants (6.91 vs. 5.52, Figure 5). This means that the foliage density of that group was higher in comparison with the control group. This also means that the amount of fresh weight mass was higher for this group (86.6 vs. 78.6 dags, Figure 4). The NAR index describes the photosynthesis intensity and defines the productivity of leaves, described by the amount of the dry mass produced per leaf surface in the time unit. The NAR index was slightly higher for the water-treated control group in comparison with consortium-treated tomato plants (0.0132 vs. 0.0114, Figure 5). Crop growth ratio (CGR), which is based on the LAI and NAR indexes, defines the amount of dry mass per soil surface taken by the plant in a specific time unit. The CGR was higher for consortium-treated plants (0.079 vs. 0.073, Figure 5), which means that that group of plants has produced more dry mass per soil surface.

### 2.4. The NGS Analysis

**Figure 6:**
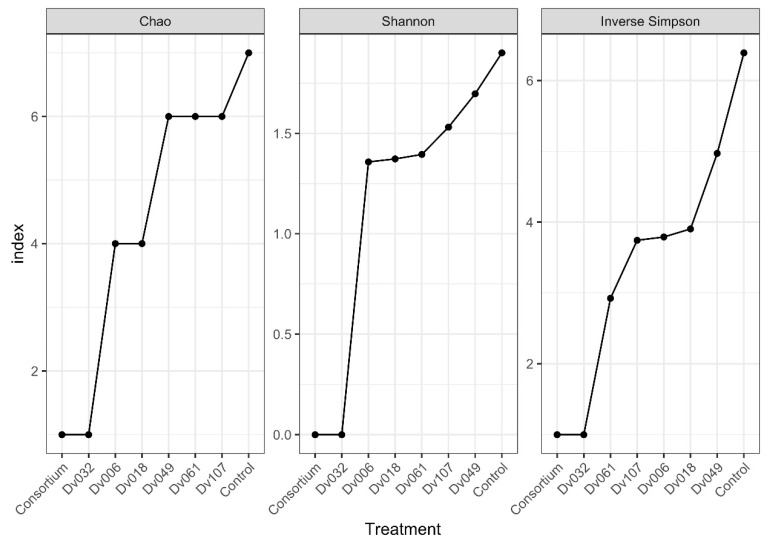
Biodiversity indexes of selected treatments based on the results of NGS analysis of the 16S rDNA region.

Obtained sequence data were submitted to the SRA database (https://submit.ncbi.nlm.nih.gov/about/sra/, accessed on 7 September 2022) under the BioProject ID: PRJNA877568, accessions: SRX17465903–SRX17465910. Alpha-diversity was verified using Chao, Shannon, and Inverse Simpson coefficients. All indexes show that the highest biodiversity occurs in the control sample (water), and the lowest biodiversity occurs for the bacterial consortium (Cons) and the strain Dv032, two treatments with the highest yield of tomato fruits (Figure 6).

## 3. Discussion

By definition, as plant growth-promoting bacteria, we consider only bacteria from the rhizosphere, rhizoplane, or endophytic and epiphytic bacteria [38]. There are several review articles concerning the plant growth-promoting bacteria (PGPB) [6,36,39]. Both rhizobacteria and endophytes are extensively studied, as they are using similar PGP mechanisms e.g.: facilitating the nutrient uptake, modulation of phytohormones level, production of antibiotics and lytic enzymes, colonization of plant surface and intercellular spaces, induction of plant systemic resistance, and reducing the effects of environmental stress [40]. However, it has been noted that the rhizosphere environment is different from that of internal plant tissues, and a variety of factors, including temperature, soil type, pH, competition for nutrients, etc., can significantly affect the plant-bacteria interaction [6]. Nevertheless, recent studies have shown that leaf and soil microbiomes are linked [41]. Some of the microbes commonly present in the soil can also exhibit an endophytic phase, which can promote insect resistance and plant growth. What is more, it was reported that insect symbionts can provide their host with beneficial functions such as the ability to suppress plant defenses or mobilize nutrients [42], and these symbionts can be acquired via the soil [41].

Many PGPB belong to well-known families like *Enterobacteriaceae*, *Pseudomonaceae,* or *Bacillaceae*, members of which have been described previously as plant growth promoting bacteria. In addition, PGPB inhabits ecological niches other than the rhizosphere and plant surface [16], and multiple plant-insect-bacteria interactions were described [36], including the role of rhizobacteria in the control of pest insects in agriculture [37]. That is why, knowing that some species within the families are closely related and have similar ecology, we decided to test if: (i) bacteria other than directly plant-associated exhibit PGP properties, and (ii) whether such bacteria can be used as ingredients of PGP bacterial (or microbial) consortia, that potentially after the formulation process could be used as biopreparates in plant production.

The perfect bioprepatate should include in its composition microorganisms exhibiting possibly many highly expressed plant-beneficial biochemical traits. Those microorganisms should also exhibit a huge biochemical potential and ability to adapt to various ecological niches. In this study, we analyzed biochemical traits, that influence plants’ root length and root extract production [39]. Bacterial isolates selected for the consortium have simultaneously highly exhibited at least two of the tested PGP biochemical traits, showed no antimicrobial activity against each other, and endured well the glycerol preservation at −80 °C. All isolates meeting those criteria have been obtained from *D. virgifera*’s digestive tract (Table 1). After identification, we confirmed that all the consortium’s species, except for *P. putida*, have been described as present in *Diabrotica balteata* Le Conte among other 24 bacterial species [43]. To our knowledge, there was no such study concerning *D. virgifera*. In another study, the authors isolated and identified 52 species of endophytic bacteria obtained from tomato roots in West Java, Indonesia. Among those species, only *P. putida* was mentioned as a tomato root endophyte [44], as well as a rice endophyte [45]. Persistence of bacteria isolated from *D. virgifera* and identified in this study, is congruent with the mentioned studies [43,44,45]. Additionally, other publications describing the tomato endophytes content [46,47] do not mention *S. liquefaciens*, *E. americana, L. garvieae, L. lactic*, *Serratia* sp., *S. plymuthica*, or *P. putida*, species isolated from *D. virgifera* digestive tract, as belonging to tomato’s natural bacterial community. The other three species constituting the consortium: *E. americana*, *L. garvieae,* and *L. lactis* are also not mentioned as tomato endophytes [44]. *E. americana* is considered an environmental bacteria with simple nutritional needs that can survive well in water [48]. *L. lactis* is a typical bacterial species of lactic acid bacterium [49], and *L. garvieae* is known as a fish pathogen [50] and as a rare opportunistic human pathogen [51]. However, all three mentioned species are able to survive in soil or water, hence are present in the natural environment [51,52,53], and according to recent studies [41,42], they could be acquired by the insects via the soil. The above information suggests that all bacterial species included in the developed consortium are commonly found in the environment, yet they are not part of the natural tomato’s microbiome. That is why the growth increase and the differences observed between tested and control tomato groups are the results of the presence of our consortium’s bacteria delivered into the soil, especially since their presence was the only factor distinguishing these two groups.

It is worth mentioning that application of the Dv032 strain produced almost the same results compared to the consortium (Figure 1). The possible explanation could be the fact that this strain is a rare case of bacterium displaying a high level of expression of several biochemical features simultaneously. That is exactly what we were aiming for when selecting the bacteria for the consortium. The Dv032 strain showed the highest level of expression of phosphatase enzyme of all 11 strains tested (Table 1). Moreover, it showed the third highest level of protease expressed as the diameter of the clear zone, the 4^th^ highest result for lipase activity (Table 1) and was one of the six strains producing ammonia. All those biochemical features are considered to be plant growth promoting. Interestingly, the DV032 strain was the only strain that had no activity of cellulase. Nevertheless, the simultaneous expression by bacterial strain of several plant growth promoting biochemical traits may be a crucial for distinguishing the bacterial strains for practical use. 

We performed an NGS analysis to assess if the treatment of tomato plants with bacteria had any influence on the composition of the tomato plant microbiome. Based on three diversity indexes (Chao, Shannon, Inverse Simpson), the highest biodiversity was observed in the negative control (Kon), which was tomato plants treated with water, and the lowest biodiversity level was observed in two treatments resulting in the highest tomato fruit yield, which was a consortium-treated and Dv032-strain-treated tomato plants. A possible explanation is the high ability of the Dv032 strain and consortium strains for colonization and environmental competition against rhizospheric bacteria. Efficient colonization of tomato plants with the tested bacterial strains might lead to reducing the natural bacterial diversity in bacteria-treated tomato plants.

As shown in the result section, the developed bacterial consortium has a significant positive effect on tomato plant growth, including the yield of fruits. Interestingly, the consortium’s bacteria promoting tomato growth are symbiotic for *D. virgifera*, a maize (*Zea mays*) pest, for which tomato plants are not a natural host. Moreover, we performed an analogical experiment on maize plants, with the very same bacteria, and received no positive effect on the maize plants’ growth (data not shown). Since it was possible to extract the bacteria from the insect’s guts, investigate them, multiply them, successfully introduce them into the soil, and obtain plant promoting effect, we conclude that the plant-promoting biochemical potential of bacteria is still underestimated.

Our results suggest that every environmental bacterium displaying the PGP qualities can be used as a potential biofertilizer, so long as it is fast-growing and able to survive in the soil. Of course, we must remember that microbial growth promoters should have a strong biosafety profile. Nevertheless, from the scientific point of view, our results provide evidence that by an aware selection of bacterial isolates, several new consortia with the desired PGP properties could be created as needed, keeping in mind that they would have to be able to act as PGP in an agricultural environment as well. Our consortium was designed to promote general plant growth, however, potentially a creation of e.g., a phosphate solubilizing rich bacteria consortium for weak soils is also possible. This approach seems to be environmentally and ecologically friendly since we did not modify the consortium’s bacteria in any way, and we used only the bacteria that are already present in the natural ecosystem. Using this approach, the multiplied bacteria were distributed in experimental cropping system without any concern for their fate. However, it was shown that soil resistance and resilience to disturbance of this kind are governed by soil physicochemical structure and that soil stability results from a combination of biotic and abiotic soil characteristics [54]. That is why the soil’s microbiome abundance and diversity are regulated by natural mechanisms, like in the case of already commonly used commercial bio-stimulants containing various microorganisms.

It is known that the insect-associated bacteria are influencing the plant reaction to pest attacks as well as insect adaptation to plant defense responses [52]. The experiments using *D. virgifera*, the most successful species in its genus were done, in which insects were treated with antibiotics to investigate if *D. virgifera*’s-associated *Wolbachia* can affect maize defense against insect attack. The results showed the down-regulation of plant defenses in the untreated insects in comparison to the antibiotic-treated and control treatments, clearly suggesting that harbored microbes can potentially mediate the down-regulation of some maize defense mechanisms through their insect hosts [55]. Contrary to non-culturable bacteria (*Wolbachia*), there is no information on the role of culturable bacteria like *S. marcescens* and *S. liquefaciens*, the main consortium’s component, in the plant host response. Our results show that the influence of the *D. virgifera*’s culturable endosymbiotic bacteria on the tomato and maize plants is different. It is unknown if the high occurrence of PGP bacteria in WCR’s gut has a special biological meaning and additional studies are needed to explain this issue. Nevertheless, the high efficiency of insects gut bacteria as plant growth promoters, in greenhouse conditions, was proved in our study. Moreover, in none other of the tested ecological niches, like endophytes (onion, cauliflower, tomato, cucumber, soy, maize, miscanthus, geranium, poinsettia, calla, primrose, and rose), insects symbionts (*Scolytidae* and *Oulema* sp.), rhizospheric and rhizoplane bacteria (rape, pea, soy, and nettle plants), dune soil, and sewage wastes (data not shown), such high occurrence, and good efficiency, of PGP bacteria, was not found, which might implicate that this may not be a singular phenomenon.

Currently, much attention is given to bacteria that colonizes the plant tissues without causing the disease symptoms but promoting the plant host growth. In our study, we tested the biochemical characteristics of the bacteria obtained from the *D. virgifera* digestive tract and found them highly effective as plant growth promoters. Those results put a matter of a PGPB in a completely different perspective and encourage us to rethink, and complete, an existing model of PGPB and plant-hosts interactions. In conclusion, our results showed that: (i) *D. virgifera* gut’s bacteria significantly promote the growth of tomato plants in greenhouse conditions, (ii) bacteria other than rhizospheric and endophytic can be considered to be plant growth-promoting, and (iii) the outcome of the assay is the base to rethink and possibly update a basic model of PGPB and plant-host interactions. It must be underlined that even though the insect gut bacteria were proven to stimulate the plant’s growth, their practical usage must be preceded by an examination of their influence on ecological safety.

## 4. Materials and Methods

### 4.1. Bacterial Strains

The experimental material constituted bacterial strains obtained from 20 adult specimens of *Scolytidae* sp., 50 specimens of cereal leaf beetle (CLB, *Oulema* sp.). and 150 specimens of western corn rootworm (WCR, *Diabrotica virgifera virgifera*). These insect species were selected for screening because they feed solely on plant tissues, and thus they digest them, potentially with an microbe aid, which would suggest high activity of insect gut-bacteria enzymes such as protease, lipase, cellulase, and phosphatase. The adult insect individuals were sacrificed, and surface sterilized by submerging in 70% ethanol for 3 min. Next, the remains of alcohol were washed using sterile distilled water (SDW). Due to the small size, CLB insects were homogenized in the mortar with SDW. In the case of WCR, the insect’s digestive tract was gently pulled out with sterile tweezers and separated from the other organs (e.g., vasa Malpighii). The extracted intestines were suspended in 1 mL of SDW and homogenized in Eppendorf’s tube using a sterile micropestle. After homogenization, 0.2 mL of each insect’s homogenate suspension was streaked on the Tryptic Soy Agar medium (Sigma-Aldrich Co., Ltd, Burlington, MA, USA) and incubated for 48 h at 27 °C. After incubation, the colonies obtained were subjected to a series of reduction streaks. Based on size, shape and color, morphologically different colonies were selected and streaked to obtain the pure cultures. Next, the pure cultures of obtained strains were preserved in −80 °C glycerol stocks for further analysis.

The plant growth-promoting properties (PGP) of each obtained strain were tested on microbiological media. Bacteria exhibiting the PGP properties were identified using biochemical and molecular methods. The bacterial consortium was created out of a group of strains displaying the highest PGP properties. The influence of bacterial consortium and each strain individually, on the growth of tomato, was tested. Each stage of the experimental procedure is described below.

### 4.2. Biochemical Strain Characterization of Bacteria

The pure cultures of all tested bacterial strains were subcultured on respective media for screening the following PGP properties: cellulase production on carboxymethylcellulose medium (CMC) [53], as a potential defense way of bacteria against soil fungi, phosphate solubility on Pikovskaya’s medium (PVK) [56], and siderophore (pyoverdine) production on succinate medium (SM), as a way to facilitate obtaining the nutrients by tomato plants, and lipase production on lipase production medium (LP), protease production on skim milk agar medium (SMA), and hydrogen cyanide production on hydrogen cyanide medium (HCN) [39], again as a way of increasing the environmental competitiveness of the consortium. After incubation, the clear zone diameter was measured (mm) for cellulase production, phosphate solubility, lipase, and protease production tests. The siderophore (pyoverdine) production ability was recorded only as presence or absence and marked as 1 or 0, respectively. Next, isolates with the highest clear zone diameter recorded in all PGP traits analyzed were additionally tested for ammonia-producing ability, and salt tolerance using concentrations of 1%, 4%, and 8% water NaCl suspension. The ammonia-producing ability was assessed only as presence (1) or absence (0).

### 4.3. Statistical Analysis

Tomato plants treated with bacteria were compared with the control sample (treated with water). The comparison was made using basic descriptive statistics and *t*-test analysis for two independent samples at α = 0.01. For the selected bacteria (11 isolates, Table 1), studies on the normality of homogeneity and variance were made. The analysis of variance ANOVA was made with the significance level of α = 0.01. The determination of homogeneous groups (post-hoc) was based on Fisher’s LSD test. Descriptive statistics in the form of an average, standard deviation (SD) and standard error of the mean (SEs) were calculated for the content of substances in bacteria. In the greenhouse experiment, the result’s statistical significance was investigated with t-Student (*p* ≤ 0.05). All statistical analyses were performed using XLSTAT (2007) Statistical Software for Excel [57].

### 4.4. Identification of Bacteria

Strains with the highest clear zone diameter recorded for all analyzed PGP traits simultaneously were identified biochemically, using the BIOLOG Gen III system (Biolog Inc., Hayward, CA, USA) according to the manufacturer’s instructions, and molecularly, by sequence analyses of the partial 16S rDNA region. The bacterial genomic DNA was isolated using standard CTAB protocol [58], and was kept at −20 °C, as a water solution, for further experiments. For identification purposes, a ~600 bp long, partial 16S rDNA sequence was obtained using TD-PCR protocol [59] and PCR primers: 16S04 (AACTCAAAGGAATTGACGG) [60] and 16S-REV [61]. The PCR products were electrophoretically separated and visualized under UV light on 1% agarose gel with the addition of MidoriGreen (Nippon Genetics Europe GmbH, Düren, Nordrhein-Westfalen, Germany) as a fluorescent dye. The electrophoresis was performed at 75 V in 40 min. in 0.5 X TBE buffer (Thermo Fisher Scientific, Waltham, MA, USA). The expected size PCR bands were cleaned using Wizard SV Gel and PCR Clean-Up System (Promega, Madison, WI, USA), and sequenced (Genomed S.A., Warsaw, Poland). Obtained nucleotide sequences were analyzed using Chromas (www.basic.nwu.edu/biotools/Chromas.html, accessed 18 February 2021, South Brisbane, Australia) and FinchTV (www.geospiza.com/finchtv, accessed 18 February 2021, Denver, CO, USA) freeware programs. The alignment was done and edited using BioEdit (v. 7.2) [62] and GeneDoc (v. 2.7.000) [63]. Finally, the sequences were analyzed using NCBI’s BlastN tool. Aligned and verified DNA sequences were submitted to the GenBank and were assigned to the individual accession numbers (Table 1).

### 4.5. Bacterial Consortium

Based on obtained results, 11 strains displaying the desired PGP properties were selected and used for the composition of the bacterial consortium. The ability of growth after glycerol preservation at −20 °C was verified. Potential antagonistic properties among strains comprising the consortium were tested using a bacterial lawn technique, as follows: an inoculum of 100 µL of 24 h 10^6^ cfu/mL Tryptic Soy Broth (TSB) bacterial suspension of the tested isolate was added to the 4 mL of cooled (50 °C) top agar (Bacto tryptone with casein 10 g (Difco), NaCl 5 g, agar 6 g), mixed and poured on the Petri’s dish with Tryptic Soy Agar (TSA) medium, and used as a base agar for the bacterial lawn. After cooling down, sterile Whatman paper discs (Ø 5 mm), each soaked with a 5 µL of 24 h culture of the tested isolate, were put on the top agar. No more than 4 isolates were tested on one plate containing the fifth isolate in the top agar. The assay was incubated at 27 °C for 48 h. After incubation, the presence or absence and size of the inhibition zone were assessed. The isolates showing any antimicrobial properties were excluded from further investigation. For the final composition of the bacterial consortium, strains displaying simultaneously a minimum of 4 out of 8 tested PGP traits were selected.

### 4.6. Preparation of Bacterial Inoculum

Selected strains were grown in the Biostat A bioreactor (Sartorius Stedim Ltd., Göttingen, Germany). The bioreactor’s inoculum comprised a 1 mL of 48 h culture of each isolate, adjusted to 6 × 10^8^ cfu/mL. Together, 11 mL of pulled 48 h cultures were added to the 1.5 L bioreactor’s medium volume, sterilely, via septum. The incubation lasted 72 h at 27 °C, with the pH set at 7.0 and oxygen level set at 25%. The rotor speed was set in a cascade mode with a minimum value of 20 rpm. The microbiological medium composition was as follows: tryptic-soy broth (TSB) (Sigma Aldrich, Burlington, MA, USA), 30 g; Nutrient Broth (Difco, Franklin Lakes, NJ, USA), 8 g; beef extract, 3 g; and bacto peptone, 5 g per 1 liter. Individual strains comprising the consortium were grown in the same way. Bacterial consortium biodiversity was assessed at the initial, and the final stage of the bioreactor’s incubation. An aliquot of 50 mL of each culture stage was sampled and centrifuged (5 min at 5000 rpm) to gently pellet the bacteria. The TSB medium (supernatant) was replaced with SDW and adjusted to 10^6^ cfu/mL. Next, 0.1 mL of each sample was serially diluted in SDW and spread on TSA medium to obtain a concentration of less than 100 cfu per plate. From such dilution for each sample, morphotypes were distinguished, identified, and their number was counted.

### 4.7. Greenhouse Test

For pot experiments, the tomato plants (v. Remiz) were grown in a greenhouse at 15–25 °C, with humidity of 30–70% and a 16/8 day/night regime. The plants were grown from seeds until they reached the height of 10–15 cm when they were inoculated with 3 mL of the inoculum of adjusted concentration measured with an optical density (OD) at 600 nm weave length (OD_600_ = 1.121), which was poured to the soil, only once, around plants base. In total, the 13 treatments were tested. The first treatment was the control group, comprising watered tomato plants. The second treatment was tomato plants treated with a bacterial consortium comprising 11 bacterial strains, and the following 11 treatments were the tomato plants treated with each bacterial strain individually. For each treatment (13) four replicates were performed, each comprising 10 plants. In total 520 tomato plants were tested. All plants grew in pots of 12 cm in diameter and 10 cm in height, filled with standard gardening soil. The plants were grown in the same conditions until the fruits appeared. Next, the tomato fruits were successively harvested until the last fruit. The number and the weight of fruits for each plant in each treatment were measured and referred to the control group. After the experiment, the survival of the tested strains was tested, by isolation and identification of bacteria from the soil samples.

### 4.8. Plant Growth Indexes Assessment

Using the results of measurement of plants length, fresh weight, and dry weight of plants, the following indexes were calculated to assess the growth of plants: (1) Leaf Area Index (LAI) [64], describing the proportion of leaves area to the area of soil taken by the plant. The plant’s surface was calculated using the DigiShape program (v. 1.5.111, CrotexNova, Rio de Janeiro, Brasil). (2) Netto Assimilation Rate (NAR), defining the productivity of leaves by showing the increase in plant dry weight per unit leaf area and unit time [65], and (3) Crop Growth Ratio (CGR), defining the amount of dry mass per a surface of soil taken by the plant in a specific amount of time [65]. Using those indexes, we assessed if the developed bacterial consortium is stimulating the growth of plants in comparison to a control group. In the second greenhouse experiment for each group (control and bacteria-treated plants), the number of fruits and their fresh weight were noted for each tomato plant separately and then summed up for the whole tested group.

### 4.9. NGS Analysis

The NGS analysis of plant DNA was performed to assess if the microbiome composition of bacteria-treated plants differs from the microbiome of the control group treated with water. We used the methodology previously described [21]. The plant DNA extraction process was verified against the presence of 16S rDNA using bacterial universal primers [66], and against the presence of commonly reported [67] contaminations in NGS projects (e.g., with *Cutibacterium acnes*) using the nested-PCR technique [68]. In total 8 samples of genomic DNA of tomato plants were analyzed. The first sample (Cons) was a genomic DNA of tomato plants treated with the bacterial consortium. The second sample (Kon) was a control sample containing the DNA of plants treated with water. The next two samples (Dv032 and Dv107) were treatments for which we observed the highest mass and number of harvested fruits, respectively. The next two samples (Dv061, Dv018) were treatments that resulted in average mass and number of fruits, and the final two samples (Dv006, Dv049) were treatments that resulted in the lowest mass and number of fruits, in comparison to the control sample (Figure 1). DNA was extracted from the pulled samples of plants from each treatment. A standard CTAB extraction protocol [58], was used to obtain genomic DNA, which was resuspended in 30 µL of 5 mM Tris/HCl, pH 8.5. DNA quality and concentration were assessed using a Nano-Drop ND-1000 spectrophotometer (Thermo Fisher Scientific), and its integrity was checked by running the 0.8% agarose gel electrophoresis. The extracted DNA was stored at −20 °C until the sequencing of 16S rRNA. The DNA extraction process was verified against the presence of 16S rDNA using bacterial universal primers [66], as well as commonly reported [67] in NGS projects contaminations (e.g., with *Cutibacterium acnes*) using the nested-PCR technique [68].

### 4.10. Bacterial Sequencing

The identification of tomato-associated bacteria was done by 16S rRNA gene sequencing at the V3-V4 hypervariable region by using Next Generation Sequencing (NGS). Sample quality control was done with Qubit dsDNA BR or HS (Thermo Fisher, Waltham, MA, USA). Separate amplicon libraries were prepared for each sample by using Quick-16S NGS Library Prep Kit (Zymo Research, Irvine, CA, USA). The sequencing was done with 20 ng of DNA per sample (MiSeq Illumina, San Diego, CA, USA) with a read length of 2 × 250 bp, an output of 25 K clusters per sample. Amplicon libraries targeting the V3-V4 hypervariable regions of the 16S rRNA gene were generated (CeGat GmbH, Tübingen, Germany). The positive and negative controls of the sequencing process were also done. After library preparation, both controls were checked and there were not any abnormalities: negative control was negative (no measurable DNA) and positive control was positive (expected DNA amount and expected fragment length).

### 4.11. Bioinformatic Processing

The reads were filtered for containing unknown nucleotides (Ns) and low-quality bases before trimming and merging using the R package DADA2 [69]. Sequences that mapped to chloroplast or mitochondrial DNA were excluded. The resulting files were used for taxonomic classification. Assigning the taxonomic labels to DNA short reads was done by examining the k-mers within a read and querying a database with those k-mers, using the Kraken2 algorithm implemented in OmicsBox software (v. 1.4.12) (Valencia, Spain). The k-mers within the tested reads were mapped to Kraken’s genomic library to the lowest common ancestor (LCA) in the taxonomic tree of all genomes that contain that k-mer. Finally, the set of LCA taxa that correspond to the k-mers in a read was analyzed to create an operational taxonomic unit (OTU) (OmicsBox, Valencia, Spain, http://manual.omicsbox.biobam.com/user-manual/module-metagenomics/taxonomic-classification/, accessed on 28 March 2022). In our study, Kraken2 results were filtered using a confidence threshold of 0.05 (OmicsBox, Valencia, Spain, www.biobam.com/omicsbox, accessed on 28 March 2022). For statistical analyses, the singletons, defined as the taxa observed in less than 2 samples, were excluded.

### 4.12. Biodiversity Analysis

Bacterial biodiversity was assessed. Alpha biodiversity, which describes the richness and equality of the microbial community in the sample was assessed using the Shannon index of dissimilarity [70]. Permutational Multivariate Analysis of Variance (PERANOVA) [71] available in a vegan package [72] was performed for beta diversity, which allows for the determination of significant differences in the level of biodiversity measured by the beta coefficient. The distance matrices needed for the procedure were calculated using the Chao similarity index [73]. The obtained p-values were calculated in a permutational procedure with 1000 permutations. All statistical analyses were carried out using R software 3.6.2 [74]. All visualizations were prepared with ggplot2 package [75].

## 5. Conclusions

Our results showed that (i) *D. virgifera* gut’s bacteria significantly promote the growth of tomato plants in greenhouse conditions, (ii) bacteria other than rhizospheric and endophytic can be considered as plant growth-promoting, and (iii) the evidence of the plant growth promoting role of the insect-gut bacteria is the base to rethink and possibly update a basic model of bacteria-plant-insect interactions.

## Figures and Tables

**Table 1:** Summary of the biochemical characterization of the isolates selected for the tested bacterial consortium. Diameters data are the mean of three replications.

No	Isolate GenBank Accession No	Identification Result	Clear Zone Diameter in mm												Positive (1)/Negative (0) Reaction			Salt tolerance (NaCl) %
			Cellulase			Phosphatase			Lipase			Proteinase			Siderophore (Pyoverdine)	Hydrogen Cyanide	Ammonia	
			Mean	SD	SEs	Mean	SD	SEs	Mean	SD	SEs	Mean	SD	SEs				
1	Dv006 KX395620	*Serratia* *liquefaciens*	7.33	0.58	0.33	3.00	1.00	0.58	12.33	0.58	0.33	19.00	1.00	0.58	1	0	0	8%
2	Dv018 KX395622	*Serratia* *liquefaciens*	3.00	0.00	0.00	8.00	1.00	0.58	7.33	0.58	0.33	20.67	0.58	0.33	1	0	0	4%
3	Dv025 KX395623	*Ewingella* *americana*	1.67	0.58	0.33	10.67	1.15	0.67	9.33	1.15	0.67	11.67	0.58	0.33	0	0	1	8%
4	Dv032 KX395624	*Serratia* *liquefaciens*	0.00	0.00	0.00	12.33	2.52	1.45	10.33	0.58	0.33	17.33	0.58	0.33	0	0	1	4%
5	Dv131 KX395621	*Serratia* *Liquefaciens*	1.67	0.58	0.33	2.67	1.53	0.88	6.67	0.58	0.33	14.00	0.00	0.00	0	0	1	1%
6	Dv107 KX395616	*Lactococcus* *garvieae*	2.67	0.58	0.33	10.67	0.58	0.33	8.33	0.58	0.33	14.33	0.58	0.33	0	0	1	4%
7	Dv111 KX395617	*Serratia* sp.	3.00	0.00	0.00	10.00	0.00	0.00	13.00	1.00	0.58	13.67	1.15	0.67	0	0	0	1%
8	Dv058 KX395618	*Serratia* *plymuthica*	0.33	0.58	0.33	1.67	0.58	0.33	8.67	0.58	0.33	19.33	0.58	0.33	0	0	1	4%
9	Dv061 KX395619	*Lactococcus* *lactis*	1.33	0.58	0.33	10.67	1.15	0.67	13.33	1.15	0.67	0.00	0.00	0.00	0	0	0	4%
10	Dv048 KX395614	*Pseudomonas* sp.	8.00	1.00	0.58	7.67	1.53	0.88	0.00	0.00	0.00	0.33	0.58	0.33	0	0	0	1%
11	Dv049 KX395615	*Pseudomonas* *putida*	7.67	0.58	0.33	0.00	0.00	0.00	0.00	0.00	0.00	0.67	0.58	0.33	0	0	1	8%
LSD_0.01_			1.267			2.804			1.652			1.499			-	-	-	-

## Data Availability

The NGS sequence data were submitted to the SRA database (https://submit.ncbi.nlm.nih.gov/about/sra/, accessed on 7 September 2022) under the BioProject ID: PRJNA877568.

## Supplementary Materials

The following supporting information can be downloaded at: https://www.mdpi.com/article/10.3390/ijms232113548/s1.
