# Supplementary material for: Insect Gut Bacteria Promoting the Growth of Tomato Plants (Solanum lycopersicum L.)
Source: Int J Mol Sci. 2022 Nov 4;23(21):13548. doi: 10.3390/ijms232113548 (PMC9657159; doi:10.3390/ijms232113548)
Supplement: Supplementary file 1 [file ijms-23-13548-s001.zip › ijms-1953204-supplementary.pdf]

Supplementary materials

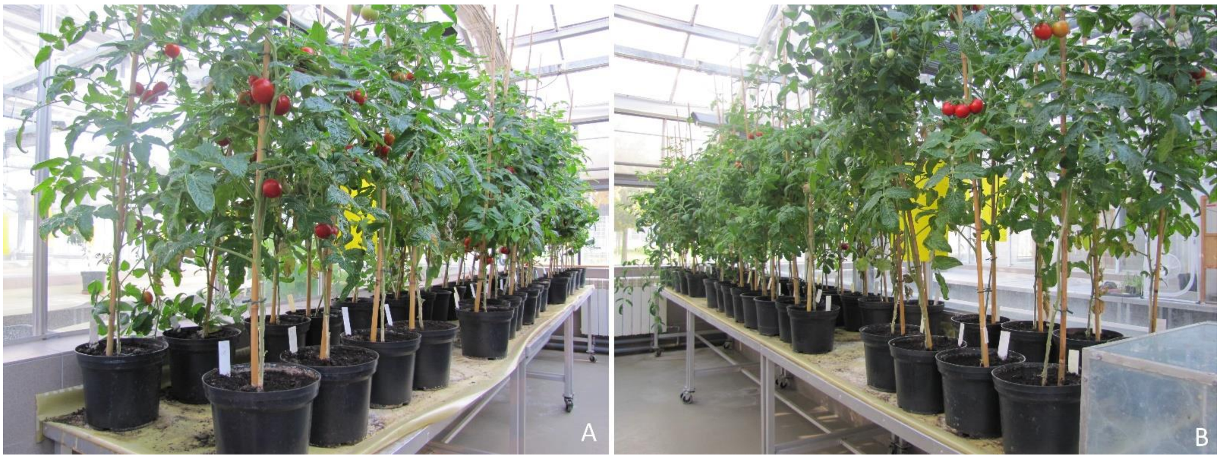

Figure S1.The phenotype of tomato plants used in the greenhouse experiment treated with the insect-gut derived bacteria (A), and the control group—not treated with bacteria (B).
